# The Pneumonias, Streptococcus, and Pneumococcus Groups

**Published:** 1918-11

**Authors:** 


**The Pneumonias, Streptococcus, and Pneumococcus Groups.** By J. G. CUMMINGS, CHARLES SPRUIT, and CHARLES LYNCH. *The Journal of the American Medical Association*, April 13, 1918.

After a conference on pneumonia, held in January, 1918, a report was drawn as to the best methods of treatment. In cases of measles patients, 291 swab cultures were made; of these 187 showed Types I and II hemolysis. In pneumonia cases it is important to note that Type I and II furnished 3 per cent. pneumonia, while Type III hemolytic streptococcus furnished 33 per cent. pneumonia cases.

It was also believed possible, after swab taking from average throats, that the streptococcus in the throat prepares the way for measles infections.

In hospital procedure, wards should be divided into three sections, in order to avoid cross infection between the carriers and the non-carriers.

First there should be a detention section for all incoming patients to be held and tested, each protected by the sheet method. If they prove carriers they should be removed into a second section, and the non-carriers into a third section. Agar plates have proved amply how necessary the sheet prevention method is.

From statistics it may be concluded that the hemolytic streptococcus is the causative organism in the complication following measles, and is the terminal cause of death in many pseudo-pneumonia cases.

Of course should vaccination prove protective, infection would be largely reduced.

The Avery method and the mouse method have been inadequate for the diagnosis of all pneumonias, and the hemolytic streptococcus in pure culture, very much after the manner of Smith, Brown and Smillie, is suggested instead.

As pneumococcus Type I infection is the only one which can as yet be treated with serum, the identification may be eliminated from consideration so far as the immediate diagnosis is concerned. The method of identification with commercial, which agglutinates in 20 minutes in a dilution of 1-160 the strain of streptococci that was isolated from post-mortem specimens, gave very good results, and was not encumbered with technical difficulties.

Where vaccination is considered, all patients should be vaccinated — carriers and non-carriers. It should be remembered that the hemolytic streptococcus is considered as the killing organism in streptococcus pneumonia, and that pneumococcus pneumonias are not as fatal as has been believed so far.

The organism is of universal prevalence. For America alone it has been isolated in various diseases in many States: in Massachusetts, in Michigan, and in California. Since it is the terminal cause of death, it seems that a specific antistreptococcus serum of a high titer applied in both groups of pneumonias, pneumococcus and streptococcus, would be the most efficient agent in reducing the present high mortality of these infections and of their complications.

There is no infection more fatal than hemolytic streptococcus septicemia. If the invasion of the streptococcus could be limited to the lung tissue, the mortality would be markedly reduced.

Universal vaccination with a triple vaccine, consisting of the hemolytic streptococcus and pneumococci Type I and II, would give favorable results, and would be a protection against complications incident to scarlet fever, as well as against the lung infections of streptococcus and complicated pneumococcus pneumonias.

